# In silico design of a multi-epitope pan vaccine targeting *Schistosoma* species

**DOI:** 10.1186/s44342-025-00053-4

**Published:** 2025-10-27

**Authors:** Daksh Gandvi, Charmi Jyotishi, Mansi Patel, Reeshu Gupta

**Affiliations:** 1 Parul Institute of Applied Sciences, Parul University, Post Limda, Waghodia Road, Vadodara, Gujarat 391760 India; 2 Research and Development Cell, Parul University, Post Limda, Waghodia Road, Vadodara, Gujarat 391760 India

**Keywords:** Schistosomiasis, Molecular dynamics, TLR4, Adenylate kinase, Vaccine

## Abstract

Schistosomiasis remains a significant public health burden, necessitating the development of effective vaccines against it. In this study, a multi-epitope subunit vaccine was designed against adenylate kinase 2 protein and evaluated for its potential to elicit protective immunity against three *Schistosoma* species. CTL, HTL, and B-cell epitopes were identified using immunoinformatics tools and linked using AAY and KK linkers, respectively. The 50S ribosomal protein L7/L12, a known TLR4 agonist, was incorporated as an adjuvant to enhance immune activation in the vaccine. Molecular docking and molecular dynamics (MD) simulations demonstrated a strong binding affinity between the vaccine and human TLR4, supported by low RMSD and Rg values, indicating structural stability. The negative binding energy further validated the vaccine’s potential for engaging TLR4. The immunogenic profile predicted robust activation of CD4+ and CD8+ T cells, as well as neutralizing antibody responses. These findings highlight the potential of the vaccine to stimulate both cell-mediated and humoral immunity, making it a promising candidate for further experimental validation against schistosomiasis.

**Supplementary Information:**

The online version contains supplementary material available at 10.1186/s44342-025-00053-4.

## Introduction

Human schistosomiasis, or bilharzia, is a neglected tropical disease (NTDs) caused by *Schistosoma* trematodes and affects over 250 million people. The disease is usually spread through contaminated water and causes chronic health issues. Schistosomiasis ranks second after malaria among parasitic diseases, affecting 75–76 countries, primarily in Africa, Asia, South America, and the Caribbean. In these countries, schistosomiasis is a significant health issue, but *Schistosoma* infections are rare in developed countries [1]. Intestinal schistosomiasis is caused by *Schistosoma mansoni* and *Schistosoma japonicum*, while urogenital schistosomiasis is caused by *Schistosoma haematobium*. Schistosomiasis threatens human health, and, if untreated, the disease can progress from mild symptoms to severe complications [2, 3]. Late-stage schistosomiasis can lead to liver and spleen enlargement, cirrhosis, ascites, bleeding, and death [4]. The WHO’s 2021–2030 program aims for a “schistosomiasis-free world,” with goals to control morbidity and interrupt transmission in selected regions by 2025 and eliminate human schistosomiasis by 2030 [5]. To effectively prevent and manage schistosomiasis, at-risk groups must receive extensive treatment, clean water, better sanitation, hygiene education, behavior modification, snail control, and environmental management.

Challenges in preventing schistosomiasis in endemic areas include a lack of precise data on transmission foci, delays in drug delivery, disease outbreaks, and negative perceptions of treatment [6, 7]. Although schistosomiasis control has succeeded in many countries, it remains a major public health issue in others. Effective strategies include vector control, diagnosis and treatment, environmental control, health education, clean water, sanitation, and surveillance [8]. Praziquantel is the preferred drug for schistosomiasis and is widely used in preventive chemotherapy (PCT) in Africa. Discovered in 1972, it effectively treats all *Schistosoma* species [9]. Artemether, artesunate, and mefloquine also exhibit antischistosomal activity. Praziquantel combined with artemether or artesunate provides 84% treatment and 96% prevention efficacy [10, 11].

In addition to pharmacological treatments, vaccine development for schistosomiasis is actively being explored; however, no vaccine has been approved for use to date. The complex life cycle of the parasite and the host immune response complicate vaccine development. Promising candidates include live-attenuated, DNA, and recombinant protein vaccines, which have shown partial protection in animal models [12]. The Sm-p80 subunit from *S. mansoni* has shown promise in vaccines, effectively preventing infections in mice and baboons, and may help address varying infection stages due to differential protein expression [13]. Sm-TSP-2, a tetraspanin membrane protein, is targeted by antibodies to disrupt schistosome teguments. High IgG subclass levels induced by the recombinant protein in a Phase 1b trial suggest the potential for antibody-dependent cell-mediated cytotoxicity. Although antibody functionality was not assessed, the immunogenicity and safety results are promising. Larger trials in high-exposure populations, including children, are needed to evaluate the vaccine’s efficacy and safety [14]. The rSh28GST vaccine for *S. haematobium* showed safety in a phase 3 trial in Senegal but lacked significant efficacy in preventing reinfection, possibly due to low IgA/IgE levels and the absence of IgG3 antibodies [15]. The Sm14/GLA-SE vaccine showed good safety and immunogenicity in phase 1 trials [16]. A phase 2a trial of this vaccine showed 92% seroconversion after three doses, confirming its potential for use in endemic regions [17]. Techniques such as single-cell sequencing may reveal new vaccine targets. However, undiscovered vaccine candidates and hybrid schistosome pairings in Africa, such as *S. haematobium* with *S. mansoni* or other species, require further investigation to understand their impact on vaccine development [13]. No established correlates of protection exist for schistosomiasis vaccines [12]. Although several potential vaccine candidates have been identified, they do not offer sufficient protection. Therefore, focusing on the rapid evaluation of new candidates using advanced technologies, leveraging mRNA vaccine platforms, and suitable animal models for immunological analysis and efficacy testing is essential [18]. Multivalent vaccines targeting multiple *Schistosoma* species in co-endemic areas can be tested using reductions in egg intensity (fecal or urine counts) for rapid efficacy assessment. The lack of effective drugs and vaccines highlights the urgent need for new therapeutic approaches to control schistosomiasis [19, 20].

Advancements in bioinformatics, immunoinformatics, and computational biology could help identify next-generation recombinant antigens from the completed schistosome genomes. A multi-epitope vaccine combines multiple epitopes from different antigens to stimulate B cells, cytotoxic T cells, and helper T cells. Immunoinformatics and structural biology tools improve vaccine design by identifying nonallergenic, nontoxic epitopes for broad population use, making it faster, cost-effective, and safer [21, 22]. The primary aims of immunoinformatics-based computational vaccinology are to identify immunodominant B- and T-cell epitopes within antigenic proteins, evaluate their immunogenicity, connect them using appropriate linkers, and determine their capacity to bind host cell receptors. This approach enables the design of vaccines using specific epitopes rather than entire proteins. These vaccines can trigger strong T- and B-cell responses upon exposure to pathogen-derived antigens [19, 23]. The development of an effective vaccine could contribute significantly to meeting the WHO 2030 Roadmap target of eliminating schistosomiasis as a public health concern [24].

This study aimed to identify potential pan-vaccine candidates by selecting kinase and histone proteins of three *Schistosoma* species based on their location, transmembrane status, nonallergic nature, thermal stability, and non-homology with the human genome. The most suitable candidate was adenylate kinase 2 (AK2). Reverse vaccinology was later integrated with in silico approaches to design multi-epitope, broad-spectrum vaccine candidates using computational methodologies.

## Material and methods

### Selection and retrieval of proteins to design multi-epitope subunit vaccine

The initial stage of creating an in silico multi-epitope subunit vaccine is the selection of proteins related to the disease of interest. We selected histone and kinase proteins to design a pan vaccine against three *Schistosoma* species (*S. haematobium*, *S. mansoni*, and *S. japonicum)*. Protein sequences were collected from the WormBase ParaSite database (https://parasite.wormbase.org/index.html) [25], which provides all the reported protein sequences in FASTA format. Proteins chosen as vaccine targets must be localized in the cell membrane or extracellular matrix, stable, antigenic, non-allergic, and nontoxic. These criteria were used to prioritize protein selection for successful vaccine development. To this end, the protein sequences of each species were analyzed for subcellular localization, transmembrane domains, instability index, antigenicity, allergenicity, and toxicity. The DeepLoc 2.0 server (https://services.healthtech.dtu.dk/services/DeepLoc-2.0/) was used to predict the subcellular localization of proteins. It uses a transformer-based protein language model to predict multi-label subcellular localization and provides interpretability [26]. Antigenicity was predicted using the VaxiJen v2.0 tool, which calculates antigenic scores (https://www.ddg-pharmfac.net/vaxijen/VaxiJen/VaxiJen.html). The server allows antigen classification solely based on the physicochemical properties of proteins without recourse to sequence alignment. A score > 0.5 indicated a probable antigen. Allergenicity was predicted using AllerTOP v2.1, which classifies sequences as probable allergens or non-allergens based on the transformation of protein sequences into similar, equal-length vectors by employing auto-cross covariance (ACC) (https://www.ddg-pharmfac.net/allertop_test/). Toxicity prediction was performed using the *in-silico* tool ToxinPred (https://webs.iiitd.edu.in/raghava/toxinpred/), which predicts the toxicity of protein sequences using an SVM (Swiss-Prot) and motif-based methods [27, 28]. HMMTOP version 2.0 (https://services.healthtech.dtu.dk/services/TMHMM-2.0/) was used to predict transmembrane helices. Proteins with 0 or 1 transmembrane helices were selected for vaccine development [29]. The instability index was analyzed using the ExPASy-ProtParam tool (https://web.expasy.org/protparam/). Proteins that met all these criteria were identified as the most promising candidates for the development of a successful vaccine [20]. The selected sequence was aligned using the multiple sequence alignment tool Clustal Omega (https://www.ebi.ac.uk/jdispatcher/msa/clustalo?stype=protein) with the ClustalW algorithm, comparing the protein sequences across three *Schistosoma* species.

### Selection of B-cell epitopes

Following the selection of the target protein, the next step was to identify regions within its sequence that could serve as potential vaccine epitopes. For B-cell epitope prediction, the ABCpred server (http://crdd.osdd.net/raghava/abcpred/ABC_submission.html) was used, which is based on an artificial neural network (ANN) algorithm. The amino acid sequence of the selected protein was submitted with the default parameters: a threshold value of 0.51, a window length of 16, and overlapping prediction were enabled to ensure comprehensive sequence coverage. The tool ranks the predicted epitopes based on the scores generated by a trained recurrent neural network. Epitopes with scores exceeding 0.5 were considered for further analysis related to IL-10 and IFN-γ induction. To evaluate the IL-10-inducing potential, selected epitopes were analyzed using the IL-10Pred (https://webs.iiitd.edu.in/raghava/il10pred/predict3.php). The peptide sequences in FASTA format were submitted, and the support vector machine (SVM) prediction model was selected with a threshold value of 0.3, while the other parameters were left at their default settings [30]. Additionally, the IFNepitope server was used to assess the IFN-γ-inducing capability of these B-cell epitopes (http://crdd.osdd.net/raghava/ifnepitope/). Peptide sequences were entered in a single-letter amino acid format using the hybrid prediction method, which combines motif and SVM approaches, to differentiate IFN-γ inducers from non-inducers. The server generated overlapping peptides and evaluated their capacity to stimulate IFN-γ response.

### Selection of CTLs epitopes

In addition to B-cell epitopes, the identification of cytotoxic T-lymphocyte (CTL) epitopes is essential for effective vaccine design. CTL peptides that bind to MHC class I molecules were predicted using the IEDB analysis resource, employing the NetMHCpan 4.1EL method (http://tools.iedb.org/mhci/*/*). This approach identifies 9-mer peptides that exhibit a high affinity for the most commonly occurring MHC class I alleles. Based on their predicted binding affinity, epitopes are classified as strong or weak binders using %Rank thresholds: peptides with a %Rank below 0.5% are considered strong binders, while those with a %Rank below 2% are categorized as weak binders. In this study, only epitopes classified as strong binders were considered for further analysis [31].

### Selection of HTLs epitopes

To predict helper T-lymphocyte (HTL) epitopes, the FASTA sequence of the selected protein was analyzed using the IEDB MHC class II binding prediction tool with the NetMHCpan 4.1 EL method (http://tools.iedb.org/mhcii/). All reference sets of human HLA class II alleles were selected, and the default parameters were maintained, with the peptide length set to 14 amino acids. The NetMHCpan algorithm classifies predicted peptides based on binding affinity into strong and weak binders using percentile rank thresholds. Epitopes with a %Rank below 0.5% were considered strong binders, whereas those below 2% were categorized as weak binders. In this study, only peptides that met the criteria for strong binding were selected for further evaluation [32].

### Antigenicity, allergenicity, toxicity, and overlapping epitopes

The predicted B-cell and T-cell epitopes were further analyzed for their antigenicity, allergenicity, and toxicity to ensure their suitability for inclusion in the final multi-epitope vaccine construct using the same tools previously employed for epitope evaluation, such as VaxiJen v2.0, AllerTOP v2.0, and ToxinPred, respectively. Epitopes identified as probable nonallergens were selected to minimize the risk of adverse immune reactions. Additionally, all selected epitopes were screened against the protein sequences of *S. haematobium*, *S. mansoni*, and *S. japonicum* to identify overlapping epitopes shared by the three species. Multiple sequence alignments were performed using the Clustal Omega tool to confirm the conservation of these epitopes. Overlapping epitopes identified through alignment were re-evaluated for antigenicity, allergenicity, and toxicity to ensure their suitability for vaccine inclusion.

### Multi-epitope subunit vaccine construct and assembly

To design the multi-epitope subunit vaccine, selected B-cell, HTL, and CTL epitopes were linked together using appropriate linkers to ensure proper separation and immunogenicity. To ensure proper folding and functional separation between the adjuvant and the first epitope, a rigid EAAAK linker was used, which promotes a helical structure and minimizes unwanted interactions. GPGPG linkers were employed between HTL epitopes to enhance epitope presentation by MHC class II molecules and promote effective T-helper responses. AAY linkers were chosen to connect CTL epitopes and bridge CTL with HTL epitopes; they support efficient proteasomal cleavage and TAP transport, thereby aiding MHC class I presentation. Finally, KK linkers were used between B-cell epitopes and to link B-cell epitopes with CTL epitopes, facilitating structural flexibility and improving epitope recognition by B-cell receptors. These linkers were selected based on their immunological compatibility and proven performance in multi-epitope vaccine design to ensure epitope integrity, independent folding, and efficient immune processing [33].

### Antigenic, allergenic, toxic potential of the final vaccine construct and physicochemical properties

The antigenicity, allergenicity, and toxicity of the final multi-epitope vaccine construct were assessed using the same tools previously used for epitope evaluation. The ExPASy ProtParam server was used to evaluate its physicochemical properties. The parameters analyzed included molecular weight, theoretical isoelectric point (pI), amino acid composition, extinction coefficient, instability index, aliphatic index, GRAVY score, and estimated half-life. These evaluations were conducted to determine the construct’s stability and suitability for further expression and development [33].

### Secondary structure prediction

The 2D structure was predicted using the PSIPRED 4.0 Workbench (https://bioinf.cs.ucl.ac.uk/psipred/). This web server evaluates the secondary structure of the vaccine and provides information on *α*-helices, *β*-strands, and coils. This server provides residue configuration, including structural alignment and interactions [34].

### Tertiary structure prediction

The three-dimensional (3D) model was predicted using the Iterative Threading Assembly Refinement (I-TASSER) server (https://zhanggroup.org/I-TASSER/). The I-TASSER server utilizes structural templates via multiple threading for protein modelling, and atomic models are built iteratively using fragment templates. The server provides the top five models along with parameters such as the C-score, estimated TM score, and RMSD value. We selected the protein 3D models with the highest C-scores. A model with high confidence can be determined using a higher C score. After employing I-TASSER to obtain the protein’s three-dimensional structure, the structure was refined using the GalaxyRefine server (https://galaxy.seoklab.org/cgi-bin/submit.cgi?type=REFINE). The server generated the five best-refined structures, which were evaluated based on GDT-HA, RMSD, clash score, MolProbity score, and the percentage of residues in Ramachandran-favored regions. The refining tool increases the structure quality by optimizing the clash score.

### Validation of tertiary structure

The last step in structure prediction was the validation of the predicted refined structure using the SAVESv6.1 tool (https://saves.mbi.ucla.edu/) [35]. The ERRAT tool on SAVESv6.1 assessed the overall quality factor of the highly refined model between 1 and 100 by analyzing the outcomes of interactions between many atom varieties that are not attached. Stereochemical quality was assessed by analyzing the residue and overall structure geometry using PROCHECK on SAVESv6.1. PROCHECK provides favored, allowed, additional allowed, and disallowed residues of amino acids and creates a Ramachandran plot, which confirms the quality, stability, and validation of the modelled structure.

### Molecular docking

Molecular docking was performed between TLR4 and the vaccine construct using the ClusPro tool (https://cluspro.org/tut_dock.php). ClusPro exhibits unparalleled precision in protein–protein docking and surpasses the performance of other comparable platforms by leveraging advanced algorithms for enhanced conformational sampling and scoring efficacy [36]. The vaccine underwent diverse docking simulations to assess its binding avidity to TLR4 (PDB ID: 4G8A). ClusPro generates an array of conformations to depict potential protein–protein docking orientations.

### Molecular dynamic simulation

Molecular dynamics simulations were performed using GROMACS version 2022.5 using the CHARMM force field. The default settings of the system were used for solvation, additional ions, and energy minimization. The root-mean-square deviation (RMSD), root-mean-square fluctuation (RMSF), radius of gyration (Rg), and solvent-accessible surface area (SASA) were calculated throughout the 300-ns simulation. For a detailed conformational analysis, the trajectories were re-indexed to separately evaluate the TLR4 receptor and vaccine construct within the complex. The RMSD, RMSF, and Rg were computed individually for each chain using GROMACS tools. This chain-wise analysis enabled the assessment of localized flexibility and structural stability across the complex. The binding free energy was calculated using the PRODIGY server (https://rascar.science.uu.nl/prodigy/) [37].

### In silico immune simulation

Immunological simulations were performed using the C-ImmSim server (https://kraken.iac.rm.cnr.it/C-IMMSIM/index.php). This server utilizes machine learning-based position-specific scoring matrices (PSSMs) for epitope prediction and performs in silico immune interaction simulations using C-ImmSim [38]. Thus, we administered three injections containing 1000 vaccine proteins without LPS, along with 100 adjuvants, and evaluated their effects at 1, 84, and 168 h. The simulation was conducted in 1000 steps using a random seed of 12345 and a simulation volume of 10. The selected injection time points (1, 84, and 168) corresponded to the typical primary and booster immunization schedules in vaccine protocols. The first dose (day 1) initiates the primary immune response, whereas the second and third injections (days 84 and 168) act as booster doses to reinforce immunological memory and enhance antibody titers. This timing was chosen based on standard vaccine schedules to model a realistic immune priming and recall-response pattern. The dynamic motions of important immune cells, such as natural killer cells, B lymphocytes, dendritic cells, macrophages, epithelial cells, CD4 HTLs, CD8 CTLs, and cytokines, were demonstrated using immune simulations.

### Codon adaptation and in silico cloning

The amino acid sequence of the final vaccine construct was reverse-translated and codon-optimized using the Java Codon Adaptation Tool (JCAT) (https://www.jcat.de/). This tool was employed to enhance the expression efficiency in a bacterial host by adjusting the codon usage to match that of *Escherichia coli* K12. The codon adaptation index (CAI) and GC content were calculated during the optimization process. A CAI value close to 1.0 and GC content between 30 and 70% are considered optimal for high-level expression. The optimized nucleotide sequence was intended for expression in *E. coli* K12, a commonly used prokaryotic expression system [39, 40]. *In silico* cloning was performed using the SnapGene software (https://www.snapgene.com/). The codon-optimized vaccine sequence was inserted into the *pUC19* plasmid vector using the *BamHI* and *HindIII* restriction sites. This step was performed to simulate the potential expression of the construct in a prokaryotic host system.

## Results

The current study revealed the best potential vaccine candidate employing computational and reverse vaccinology approaches targeting kinase and histone proteins of *S. haematobium*, *S. japonicum*, and *S. mansoni*.

### Reverse vaccinology pipeline to identify suitable vaccine candidate

The amino acid sequences of 397 kinases and 63 histone proteins from *S. haematobium*, 188 kinases and 54 histone proteins from *S. japonicum*, and 420 kinases and 63 histone proteins from *S. mansoni* were retrieved from the WormBase database in the FASTA format. Among these proteins, AK2 was identified as a potential vaccine candidate using an integrated screening pipeline. This protein is localized to the cell membrane and possesses a single transmembrane domain. Additionally, physicochemical analysis demonstrated the stability of the protein, as indicated by a low instability index (39.91) and high antigenicity (0.5948). Furthermore, AK2 was predicted to be nonallergenic and nontoxic, supporting its potential safety and immunogenicity as a vaccine target. Based on these attributes, AK2 was considered a suitable candidate for vaccine development. Therefore, the amino acid sequences of AK2 for *S. haematobium*, *S. japonicum*, and *S. mansoni* were downloaded from the UniProt database (*S. haematobium*: A0A922IIX1, *S. japonicum*: C1LK75, and *S. mansoni*: A0A3Q0KGQ7).

### B-cell epitope prediction

B-cell epitopes play a crucial role in enhancing antigen-specific antibody production and memory cell formation. The development of epitope-based vaccines relies on the accurate identification of B-cell epitopes within the selected protein. Using the ABCpred server, 76 B-cell epitopes were initially predicted based on the default threshold value (0.51) of the server. These epitopes were screened for antigenicity, allergenicity, and toxicity to ensure their suitability for vaccine development. The analysis revealed that only two epitopes met the selection criteria (Table 1). Furthermore, these epitopes have been shown to stimulate IL-10 and IFN-γ production, thereby enhancing immune responses by activating macrophages and natural killer (NK) cells.

**Table 1:** B-cell epitopes for vaccine design

SN	B-cell epitopes	Antigenicity	Allergenicity	Toxicity	IFN-γ	IL-10 inducer
1	VPMNWSTDLSNESSVM	0.8825	No	No	Positive/3	Inducer/0.485
2	IVVAHFANDIVRLLTT	0.6263	No	No	Positive/5	Inducer/0.528

### CTL and HTL epitope prediction

Cytotoxic T-lymphocytes (CTL) epitopes with 9–10 amino acids with the most frequent MHC-I have been identified deploying the Immune Epitope Database (IEDB) NetMHCpan 4.1 EL approach. A total of 244 epitopes were initially predicted based on the default IC50 value < 500 nM and percentile rank (< 0.5) of NetMHCpan 4.1 EL. These epitopes were screened across all three *Schistosoma* species to identify overlaps, resulting in the selection of 100 epitopes. These overlapping epitopes were assessed for antigenicity, allergenicity, and toxicity to ensure their suitability for vaccine development. Following this screening, five epitopes were identified as MHC class I binders, making them strong candidates for eliciting a cell-mediated immune response. Therefore, these five epitopes were selected for vaccine development (Table 2).

**Table 2:** List of cytotoxic T-cell epitopes in adenylate kinase 2

Sr. no.	CTL epitope	Length	Allele	Antigenicity	Allergenicity	Toxicity	Peptide score	Percentile rank
1	TTESTFIGR	466–474	HLA-A*68:01	1.2213	No	No	0.924816	0.05
2	TESTFIGRVF	467–476	HLA-B*44:03	2.1062	No	No	0.782543	1.0
3	RLLTTGSFHF	408–417	HLA-B*15:01	0.5235	No	No	0.454071	0.37
4	KSSIVVAHF	394–402	HLA-B*58:01	1.2253	No	No	0.970546	0.02
5	FTQFGVKGV	476–484	HLA-A*68:02	0.8172	No	No	0.498324	0.19

The same IEDB tool was used to predict MHC class II epitopes, yielding a total of 125 predicted sequences. The selected epitopes were further examined for overlapping patterns across the three *Schistosoma* species, resulting in the selection of 62 epitopes. These 62 epitopes were screened for antigenic potential, allergenicity, and toxicity to predict their suitability for vaccine development. Of the predicted candidates, four MHC class II binding epitopes were chosen for vaccine construction based on their high antigenicity, percentile ranks below 2.0, and confirmed nonallergenic, nontoxic properties (Table 3).

**Table 3:** List of HTL in adenylate kinase 2

Sr. no.	HTL epitope	Length	Allele	Antigenicity	Allergenicity	Toxicity	Peptide score	Percentile rank
1	AHFANDIVRLLTTG	400–413	HLA-DRB3*01:01	0.7194	No	No	0.3873	1.2
2	QSKIYNDLATVFCN	504–517	HLA-DRB3*01:01	0.5499	No	No	0.5585	0.65
3	SVFEVDIVIYIEER	255–268	HLA-DRB3*01:01	0.9638	No	No	0.4963	0.79
4	VDIVIYIEERSVPM	259–272	HLA-DRB1*15:01	0.7206	No	No	0.7396	0.53

Population coverage analysis using the IEDB tool revealed that the current CTL/HTL epitope set provides limited coverage in endemic African regions, averaging 33.41% (range 29.95–37.62%). Across global regions, the highest class combined coverage was observed in North America (44.69%), East Asia (41.73%), and Europe (40.49%). In contrast, Central America exhibited the lowest coverage (5.72%). The worldwide population coverage was estimated at 38.32%. The overall mean coverage across all regions was 31.35% (SD ± 11.08), indicating substantial variability between geographical regions.

### Construction of multi-epitope subunit vaccine

The multi-epitope vaccine was developed by integrating the selected epitopes to enhance immune activation. The 50S ribosomal protein L7/L12 (P9WHE3.1), a TLR4 agonist, was used as a supplement to enhance the immune response. The vaccine construct included six HTL epitopes, eight CTL epitopes, and three B-cell epitopes, linked using GPGPG, AAY, and KK linkers, respectively. To ensure a contiguous sequence in the final design, the adjuvant was linked to the epitopes using an EAAAK linker. The final vaccine construct consisted of 399 amino acids, with epitopes linked using these specific peptide linkers to maintain structural integrity and enhance immunogenicity.

### Evaluation of antigenicity, allergenicity, toxicity, and physicochemical analysis of vaccine constructs

The antigenic score of the vaccine was 0.5904, which confirms its antigenic nature. Allergenicity prediction using the AllerTOP v2.1 server classified the vaccine as a non-allergen, whereas toxicity assessment via the ToxinPred server confirmed that the protein was nontoxic. Physicochemical analysis demonstrated that the vaccine exhibited good thermostability (aliphatic index = 93.71) and hydrophilicity (GRAVY score = 0.221) (Table 4). These findings suggest that the designed vaccine construct has favorable properties for initiating an immune response in the host. The secondary structure of the multi-epitope vaccine construct was comprised of approximately 52.19% *α*-helices, 17.09% *β*-strands, and 30.71% coils. The high proportion of *α*-helices suggests good structural stability, whereas the moderate content of coils may provide the necessary flexibility for proper epitope presentation. These structural features are favorable for maintaining overall protein integrity and facilitating effective immune recognition.

**Table 4:** Evaluation of vaccine design

Number of amino acids	399
Molecular weight	42454.67
Total number of atoms	6028
Atomic composition	Carbon (C) 1930 Hydrogen (H) 3030 Nitrogen (N) 492 Oxygen (O) 569 Sulfur (S) 7
Total number of negatively charged residues (Asp + Glu)	46
Total number of positively charged residues (Arg + Lys)	39
Formula	C_1930_H_3030_N_492_O_569_S_7_
Extinction coefficients	23380
Antigenic score	0.5904
Allergenicity	Non-allergen
Toxicity	Nontoxic
Estimated half-life	30 h
Instability index	19.27
Aliphatic index	93.71
Grand average of hydropathicity (GRAVY)	0.221

### Tertiary structure prediction

To investigate the interactions between the vaccine and host immune cells, a stable three-dimensional (3D) structure is crucial. I-TASSER was used to generate 3D models of the vaccine construct, and the resulting structures were assessed for structural stability. Five models were generated based on the optimal threading templates from the PDB library, including 2acxA, 2bcjA, 3c4wB, 3c51B, 3nynA, 4myiA, 4tnbA, 4yhjA, 7t4tA, and 8em8A. Each model was assigned an individual C-score with values of −1.72, −1.88, −2.97, −4.39, and −3.42. The model with the highest C-score, a root-mean-square deviation (RMSD) of 10.8 ± 4.6 Å, and a TM-score of 0.51 ± 0.15 was selected.

**Fig. 1:**
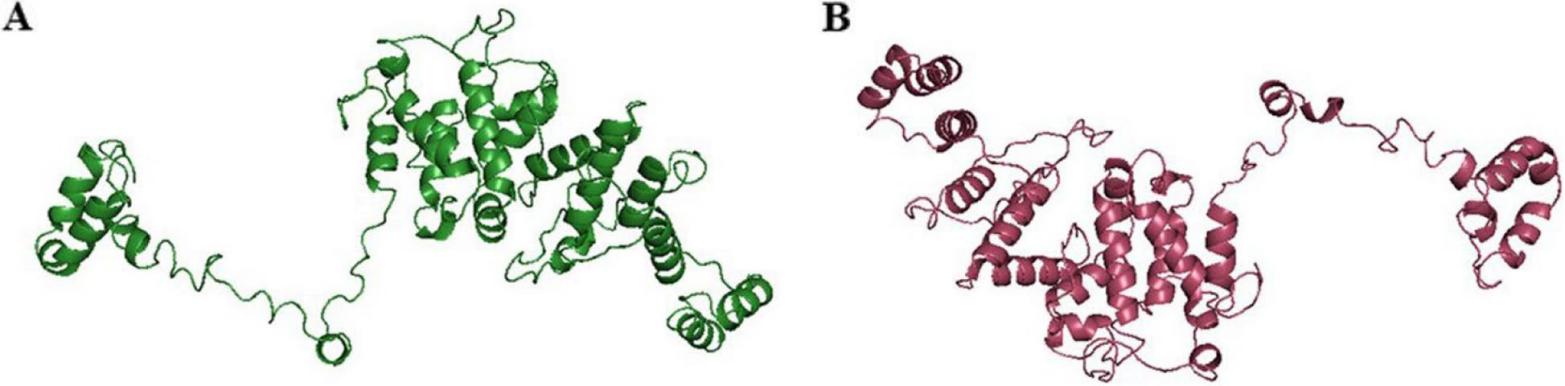
**A** Three-dimensional structure of the vaccine by I-TASSER. **B** Refined 3D structure of the vaccine constructs using the Galaxy Web server. Model 4, Rama favored = 90.7%, clash score = 12.3, *RMSD* = 0.540, and *GDT-HA* = 0.9029

To enhance the structural accuracy, the chosen 3D model was refined using the GalaxyRefine server, which optimized the clash score and improved the structural quality. Five refined structures were generated from the initial model for further analysis. Compared with the original model, the five refined models from GalaxyRefine showed improved structural parameters. Model 2 demonstrated the most significant improvements, with a Rama-favored value of 90.7%, RMSD of 0.531, poor rotamers of 0.3, GDT-HA of 0.9091, clash score of 11.8, and MolProbity score of 2.115 (Table 5). This model was selected for further analysis (Fig. 1).

**Table 5:** Characteristics of the refined structures of original model of vaccine

Model	GDT-HA	RMSD	MolProbity	Clash score	Poor rotamers	Rama favored
Initial	1.0000	0.000	2.868	4.2	12.6	71.0
MODEL 1	0.9135	0.523	2.106	11.3	0.3	90.4
MODEL 2	0.9091	0.531	2.115	11.8	0.3	90.7
MODEL 3	0.9029	0.540	2.131	12.3	0.3	90.7
MODEL 4	0.9085	0.543	2.188	13.5	0.3	89.9
MODEL 5	0.9179	0.519	2.117	11.7	0.3	90.4

**Fig. 2:**
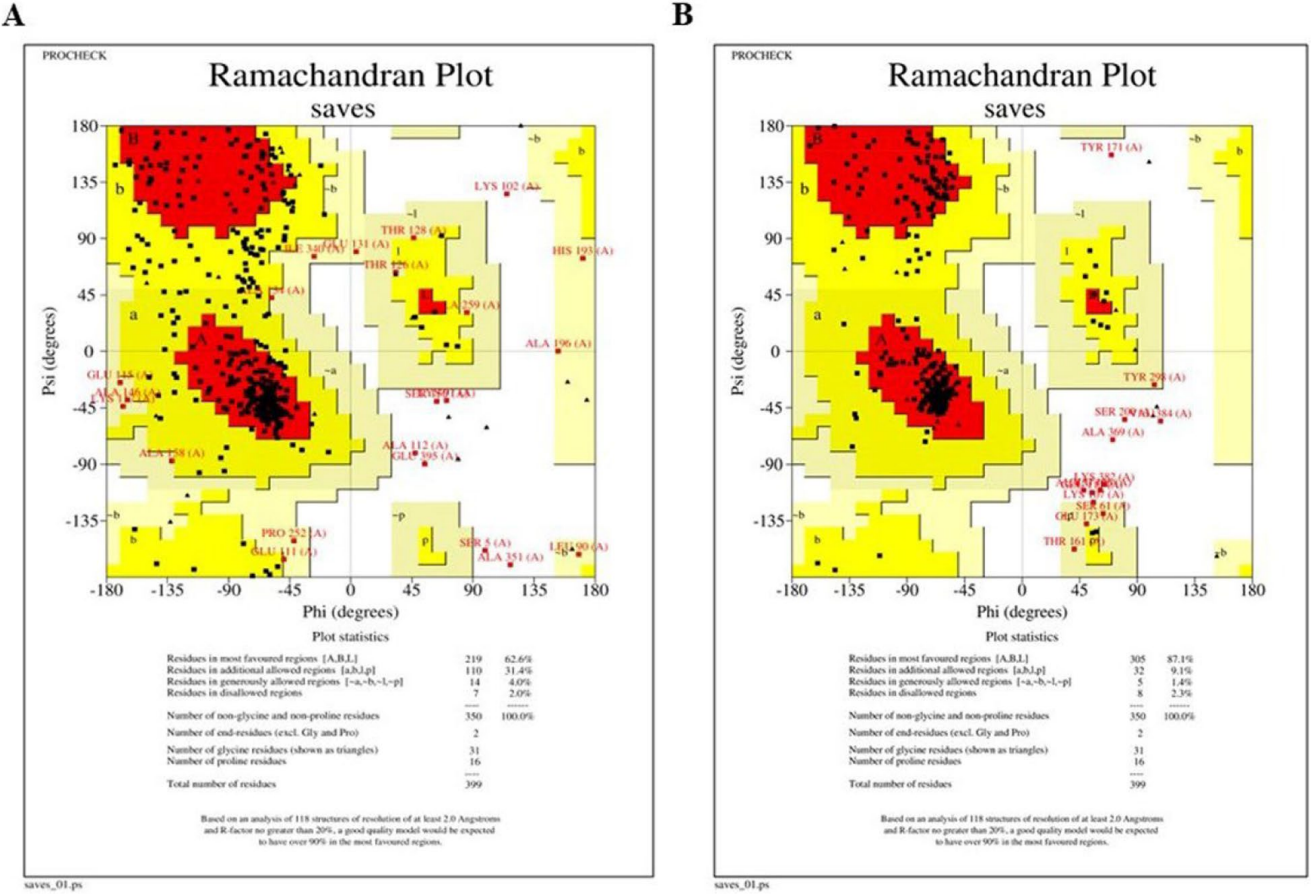
Ramachandran plots of **A** pre-refined and **B** refined tertiary structure obtained using the SAVESv6.1 server

The pre-refined structure contained 62.6% of residues (219 amino acids) in favored regions, 31.4% (110 amino acids) in allowed regions, 4% (14 amino acids) in generously allowed regions, and 2% (7 amino acids) in disallowed regions (Fig. 2A). The five refined models were further validated using the SAVESv6.1 server to assess their structural qualities. The ERRAT analysis yielded an overall quality factor of 88.204, confirming the reliability of the model. PROCHECK evaluation showed that 87.1% of residues (305 amino acids) were in favored regions, 9.1% (32 amino acids) in allowed regions, 1.4% (5 amino acids) in generously allowed regions, and 2.3% (8 amino acids) in disallowed regions (Fig. 2B). These findings indicate that the predicted 3D structure of the vaccine construct is of high quality, making it suitable for further analysis.

### Molecular docking and molecular dynamics simulation

**Fig. 3:**
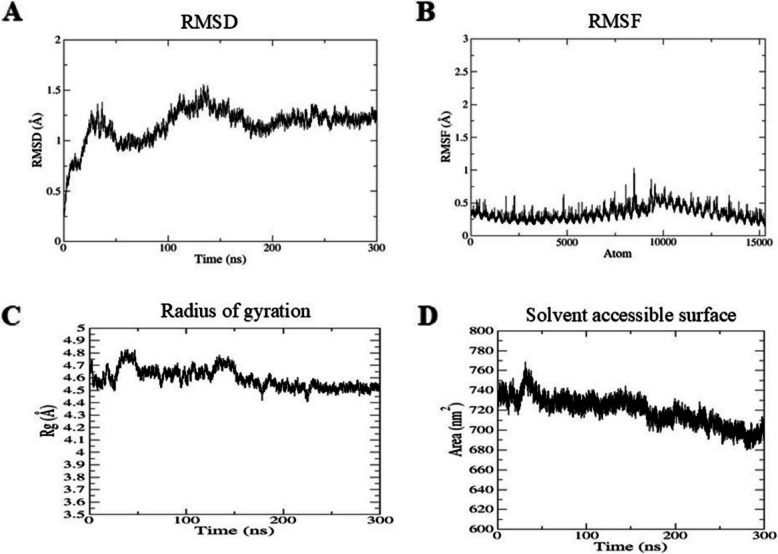
Molecular dynamics simulation study of TLR4-vaccine construct. **A** Root-mean-square deviation. **B** Root-mean-square fluctuations. **C** Radius of gyration. **D** Solvent-accessible surface area for a time duration of 300 ns

Docking analysis revealed that the vaccine construct was in close contact with chain A of TLR4 and formed polar interactions with TLR4 (Table 6). Energy minimization confirmed the structural stability of the vaccine construct, achieving a final potential energy of −15,188,023 ± 4443.45 kJ/mol after 6229 steps. These values indicate a relaxed and sterically acceptable initial conformation for downstream MD simulations (Figs. 3). The vaccine-TLR4 complex’s stability and compactness were assessed using the RMSD and Rg, as shown in Fig. 5. The RMSD remained below 1.5 nm, with an average of 1.159 ± 0.0009 Å (Fig. 3A), indicating a stable structure. Additionally, the root-mean-square fluctuations (RMSF) were calculated over 300 ns of simulation to analyze residue-level fluctuations (Fig. 3B). The RMSF plot showed minimal fluctuations in chain A (regions 1–5824, *RMSF*: 0.27 ± 0.0008 SEM) and chain B (regions 5825–11,689, *RMSF*: 0.41 ± 0.001 SEM) (Fig. 3B). Throughout the simulation, the average ligand RMSF remained stable at 0.40 ± 0.002 Å. The radius of gyration averaged 4.59 ± 0.001 nm, stabilizing after 30 ns, confirming the stability of the 3D protein structure during molecular dynamics (MD) simulations (Fig. 3C). Furthermore, the solvent-accessible surface area (SASA) was measured at 718.49 ± 0.084 nm^2^, demonstrating that the vaccine-TLR4 complex’s hydrophobic core continued to remain free in the surrounding aqueous environment (Fig. 3D).

**Table 6:** Residues of vaccine forming polar interaction with TLR4

SN	TLR4 residues	Vaccine residues
1	GLN-200	LYS-73
2	SER-105	GLU-355
3	THR-106	GLU-335
4	GLN-129	GLU-335
5	THR-151	ARG-218
6	ARG-460	ASN-373
7	HIS-431	VAL-368
8	GLN-489	ARG-377
9	ASN-365	VAL-398
10	THR-413	SER-397
11	LYS-362	ASN-394
12	ARG-264	ASP-391
13	LYS-362	GLU-395
14	ARG-355	GLN-294
15	GLU-425	LYS-296
16	GLN-523	GLY-270
17	GLN-547	GLY-272
18	LYS-153	VAL-333
19	LYS-153	SER-332
20	GLN-485	ARG-377
21	GLN-4366	SER-397
22	ASN-339	GLU-395
23	ARG-355	SER-295

To obtain component-level insights, we indexed the complex to separate the TLR4 and vaccine chains. The analysis, presented in Supplementary Figs. 1 and 2 and supplementary Tables 1 and 2, shows that both TLR4 and vaccine components remained structurally stable throughout the 300-ns simulation. This indicates that the vaccine construct is compatible with TLR4 and maintains conformational stability during the interaction. The RMSD plots indicate that both components stabilized after approximately 30 ns. The radius of gyration remained consistent for each chain, further confirming the overall compactness and stability.

These findings strongly support the high binding affinity of the vaccine for TLR4 during the 300-ns simulation, confirming its structural stability and potential efficacy.

The binding affinity and interfacial properties of the vaccine-TLR4 complex were analyzed using the PRODIGY tool. The predicted Gibbs-free energy (ΔG) was −8.6 kcal/mol, corresponding to a dissociation constant (K) of approximately 4.8 × 10^−⁷^ M at 25 °C, indicating a moderately strong interaction within the nanomolar range. A detailed analysis of the interfacial contacts (ICs) revealed a diverse interaction profile comprising eight charged–charged contacts, eight charged–polar contacts, and five charged–apolar interactions, reflecting substantial electrostatic and polar contributions to binding stability. In addition, the interface contained six polar–polar, eight polar–apolar, and five apolar–apolar contacts, suggesting the presence of hydrogen bonding and hydrophobic interactions. The noninteracting surface (NIS) exhibited a charged composition of 25.54%, indicating that a moderate proportion of charged residues remained solvent-exposed. These findings support the thermodynamic stability of the complex and highlight the mixed nature of the inter-residue interactions at the binding interface.

### Immune simulation

**Fig. 4:**
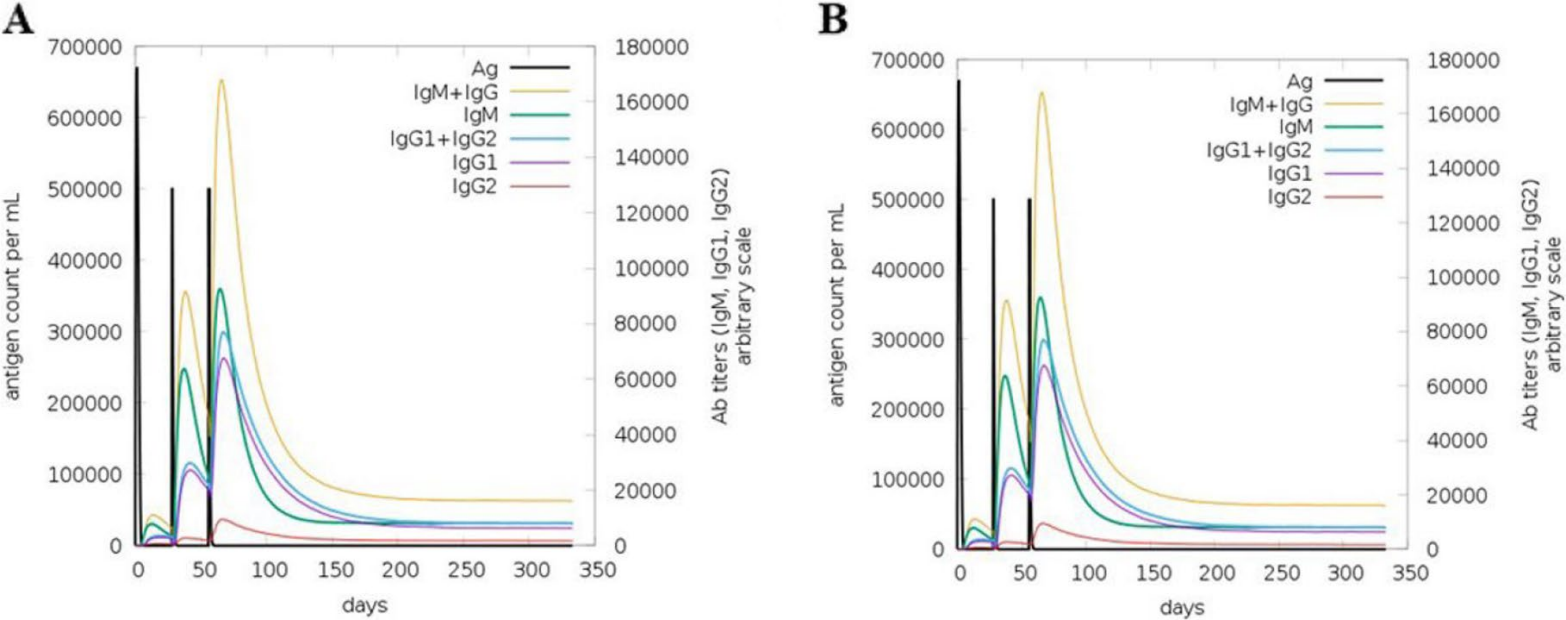
In silico simulation of the multi-epitope subunit vaccine. **A** Antigen and immunoglobulins. **B** Production of cytokines and interleukins

**Fig. 5:**
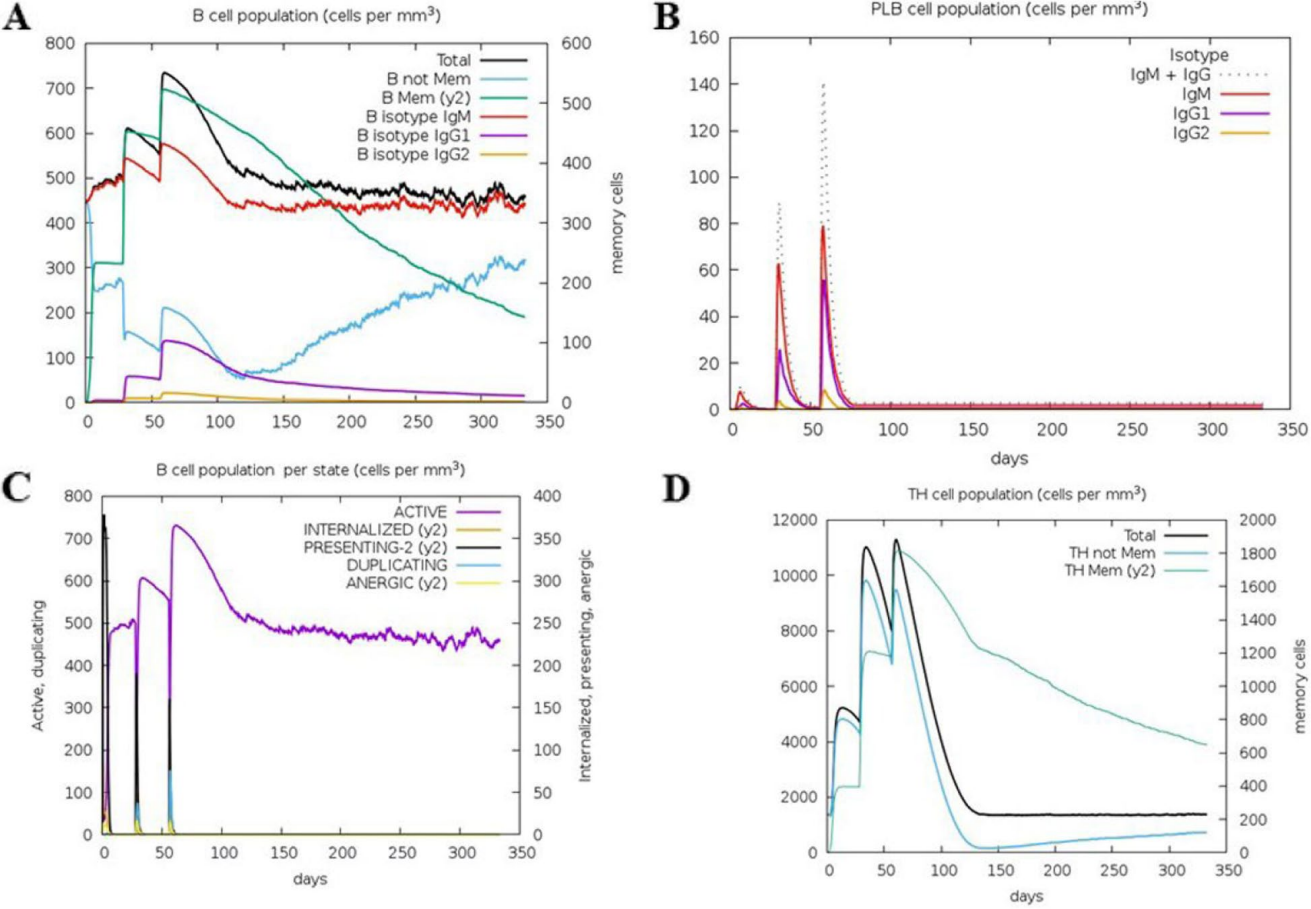
In silico simulation of the multi-epitope subunit vaccine. **A** B-cell population per stated TH cell population. **B** PLB cell population. **C** B-cell population per state. **D** Th cell population

**Fig. 6:**
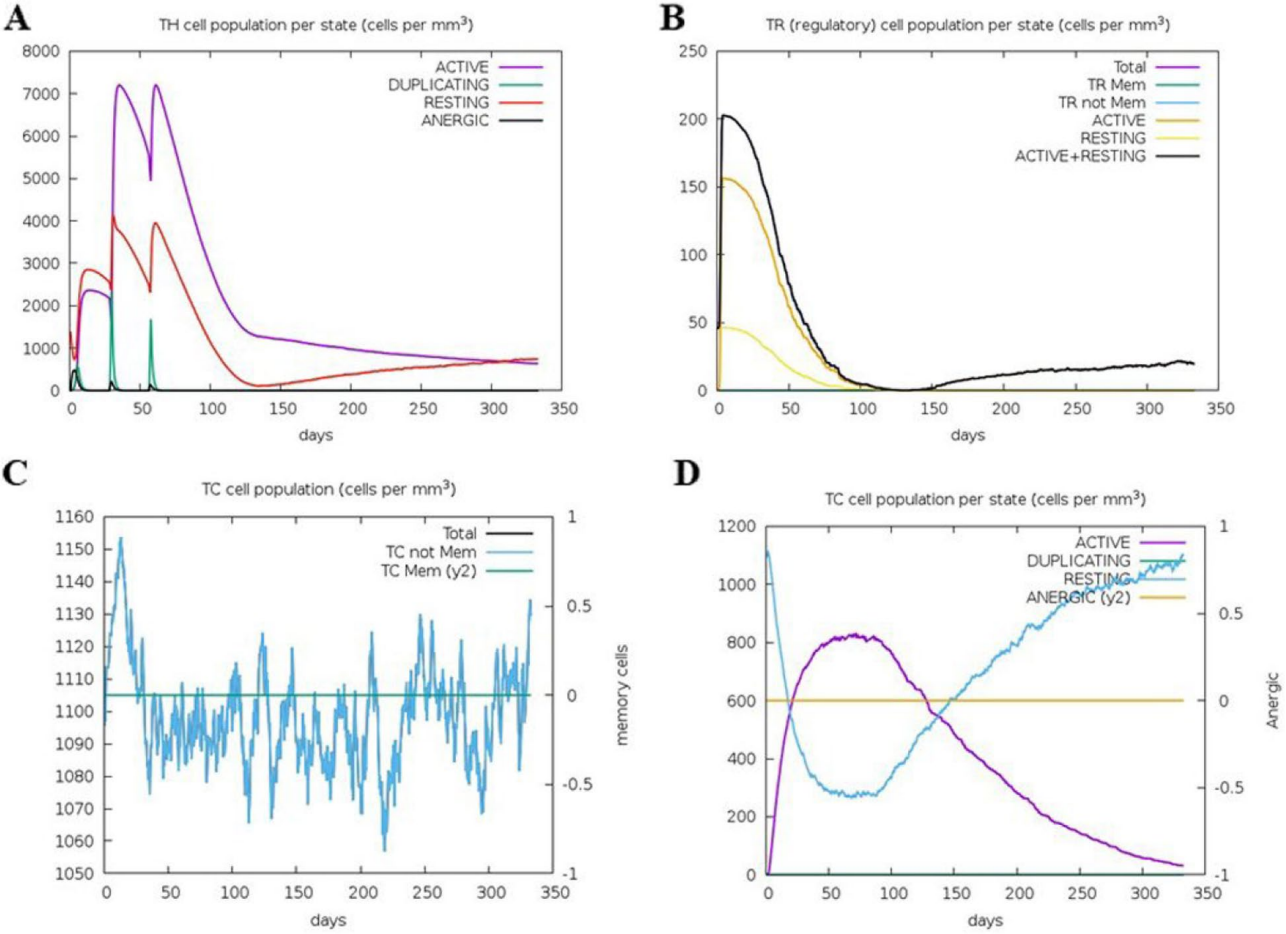
In silico simulation of the multi-epitope subunit vaccine. **A** T-helper cell population per state. **B** T-regulatory cell population per state. **C** T cytotoxic cell population per state. **D** TC cell population per state

**Fig. 7:**
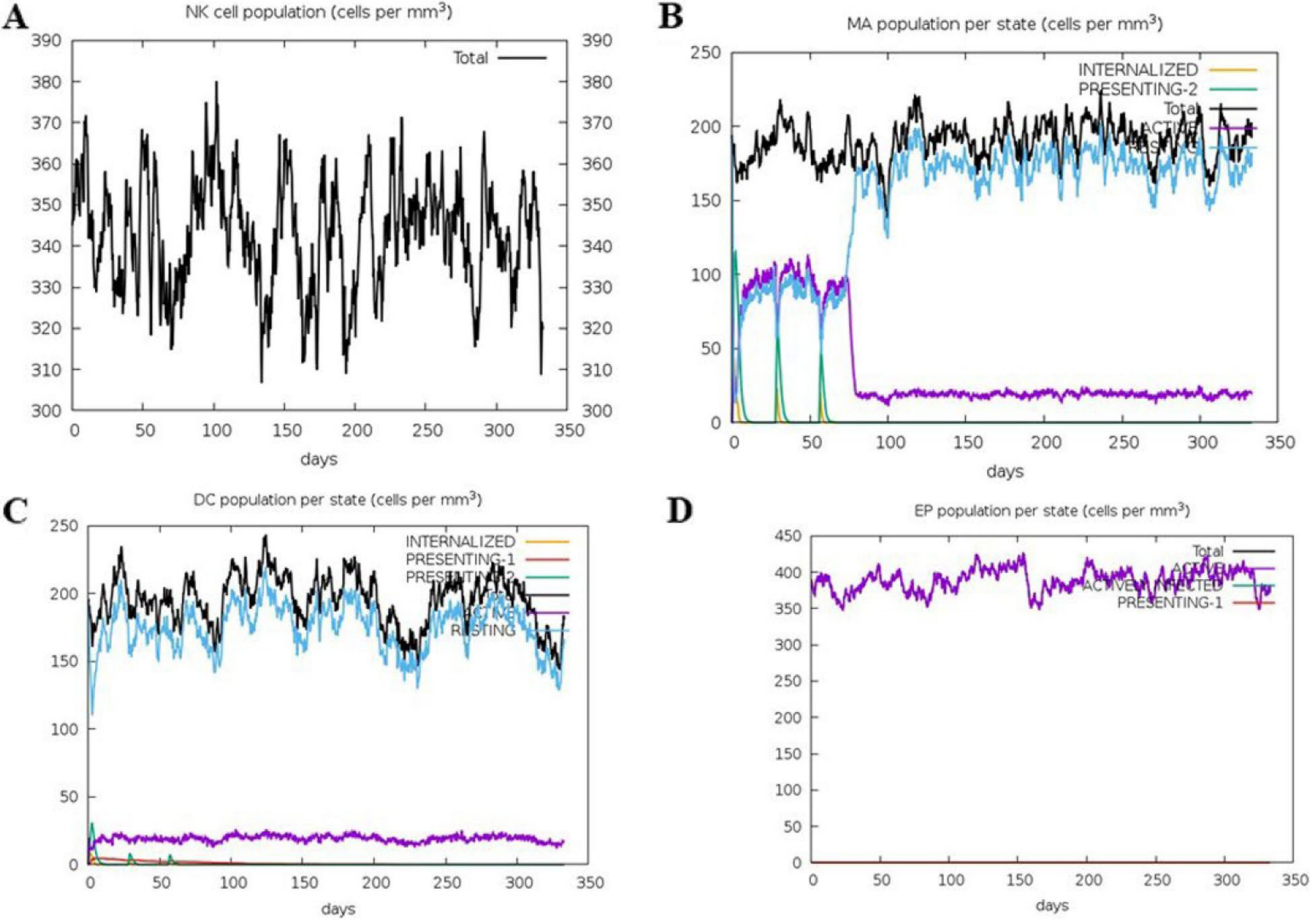
Immune cell population dynamics following vaccine simulation against schistosomiasis over 350-day period. **A** Natural killer (NK) cell population showed oscillatory behavior, indicating baseline innate immune activity post-vaccine exposure. **B** Macrophage (MA) populations across different states showed an initial peak in active and presenting cells, followed by stabilization, indicative of early antigen processing and subsequent immune regulation. **C** Early peak in presenting DCs indicates strong antigen presentation following vaccination, especially around days 10–60. **D** Epithelial cells remain stable (~380–420 cells/mm^3^) with small antigen presentation activity

Following secondary immune stimulation, IgM and IgG1 titers increased significantly, and active B-cell levels remained consistently elevated (Fig. 4A-B A and B). T-cell analysis indicated a rapid rise in Th memory (y2) and active Th cells after the first immunization, with further enhancement after the second dose. Although regulatory T cells were initially activated, their numbers rapidly declined. Active cytotoxic T (Tc) cells increased post-primary immunization, remained elevated after the second dose, and gradually decreased thereafter, whereas resting Tc cells followed the opposite trend. No changes were observed in the anergic (y2) and memory Tc cells (Figs. 5 and 6). Additionally, key immune cytokines, such as IFN-γ and IL-2, crucial for immune responses against *S. haematobium, S. mansoni,* and *S. japonicum*, were prominently secreted, demonstrating robust immune activation (Fig. 7). Following simulated vaccinations at days 1, 84, and 168, natural killer (NK) cell populations (Fig. 7A) remained relatively stable with minor oscillations, indicating sustained innate surveillance. Macrophage populations (Fig. 7B) exhibited a marked shift from active and presenting states early after each immunization to a predominance of presenting state-2, reflecting ongoing antigen processing. Dendritic cells (Fig. 7C) showed early peaks in presenting and internalized states, consistent with rapid antigen uptake and presentation following vaccination. Epithelial cell populations (Fig. 7D) remained largely constant, with a small fraction transitioning to active or presenting states, suggesting a role in maintaining mucosal immunity. These patterns collectively indicate that innate and antigen-presenting cell activation occurs in a temporally coordinated manner, without evidence of cell population collapse or contradictory trends.

### Codon optimization and in silico cloning

**Fig. 8:**
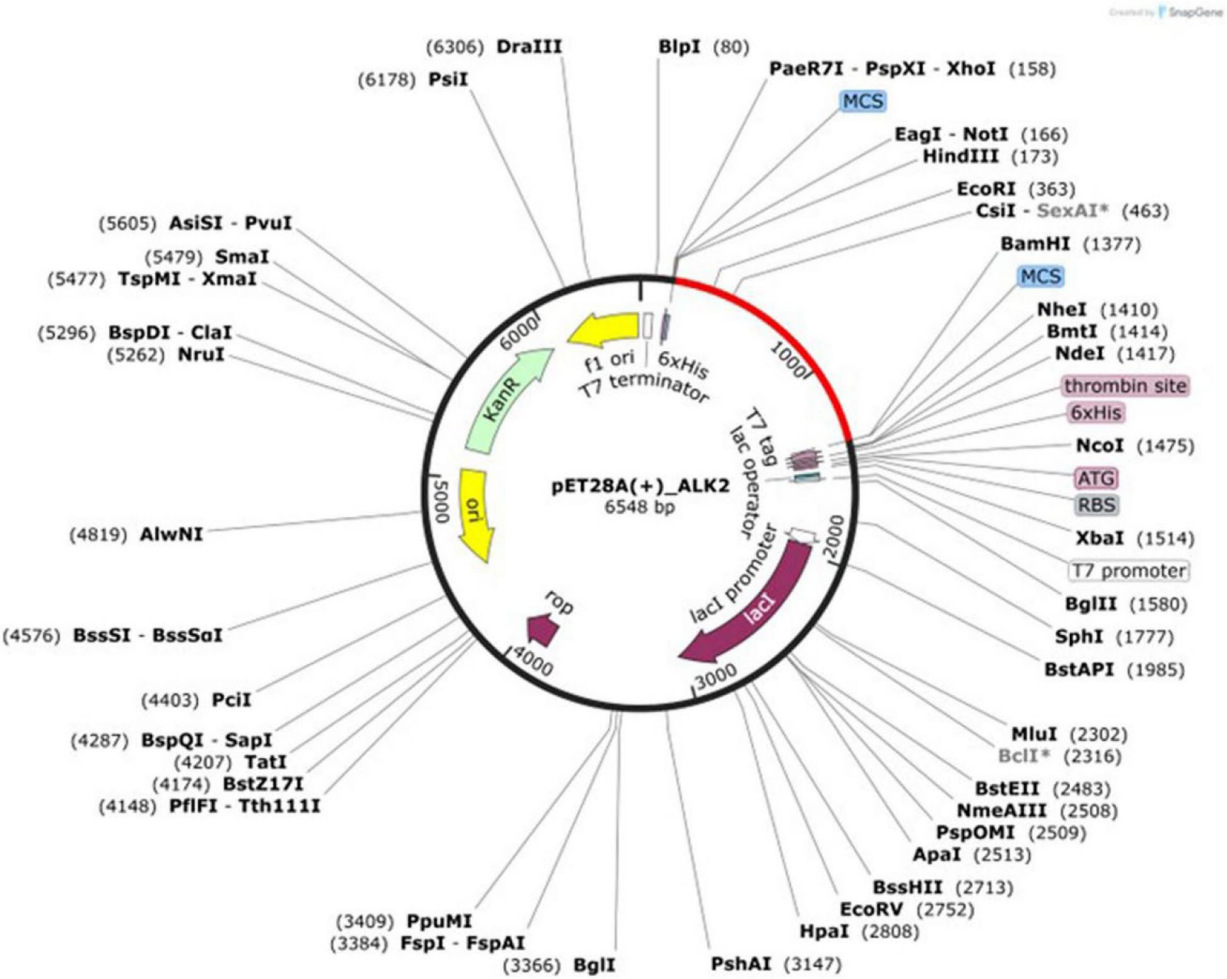
In silico cloning of vaccine into pET28a vector

The peptide sequence of the vaccine construct was reverse-translated into a DNA sequence, followed by optimization of the vaccine construct using *E. coli* K12. The optimized sequence had a predicted CAI of 1.0 and a GC content of 49.62%, compared to *E. coli’s* native GC content of 50.734%. The multi-epitope vaccine was inserted into the *pUC19* vector using *HindIII* and *BamHI* restriction enzymes to ensure its expression in *E. coli* systems. To confirm the insertion of the vaccine design, the vector was digested using the restriction enzymes *BamHI* and *HindIII*, generating a successful 6548-bp clone (Fig. 8).

## Discussion

Vaccination is a highly effective and cost-efficient strategy for the rapid control of schistosomiasis [41]. Several types of vaccines, including live-attenuated, DNA, mRNA, and recombinant protein vaccines, are currently in the pipeline for phase 1 and phase 2 clinical trials [13]. Currently, no widely recognized vaccine has been developed to prevent schistosomiasis [42]. Notably, conventional vaccine development methods are time-consuming, often taking years to develop. *In silico* multi-epitope subunit vaccine development can accelerate vaccine development research and manufacturing processes, and these vaccines improve safety, ease of administration, immunogenicity, and large-scale antibody production [43]. As a vaccine component against three *Schistosoma* species (*S. haematobium*, *S. mansoni*, and *S. japonicum*), we developed a multi-epitope subunit vaccine using an *in silico* approach. These species play vital roles in the development of schistosomiasis [44]. Similar to schistosomiasis, tuberculosis remains a major global health threat, complicated by drug resistance and limited vaccine efficacy [45]. Recent multi-epitope vaccine designs targeting conserved antigens in TB offer a promising framework that could be adapted for helminth infections, such as schistosomiasis, under the One Health approach.

We specifically chose all histone and kinase proteins as they play an important role in their growth and reproduction [46–53]. Several studies have shown that DNA methylation and histone modifications play important roles in schistosome development, reproduction, and host–schistosome interactions [46, 54]. For example, it has been shown that SmHDAC8 is involved in schistosome cytoskeleton organization [47]. Similarly, adenylate kinases influence the growth of *Schistosoma* [55]. These results suggest the role of epigenetics and kinase proteins in schistosomiasis. Identifying the kinase proteins and epigenetic alterations of schistosomes will be helpful in the development of novel medications and therapies for schistosomiasis. Therefore, different protein sequences of kinases and histone proteins were selected and retrieved from the ParaSite WormBase database. However, we did not identify any histone proteins that fulfilled the criteria of physiochemical attributes in addition to antigenicity and allergenicity. However, we identified AK2 as a suitable vaccine candidate based on its physicochemical attributes, location, antigenicity, allergenicity, and toxicity. Moreover, the protein did not contain any transmembrane domain, which is essential for the rapid production of vaccines on a larger scale. Our analysis supports previous data, where immunization with adenylate kinase proteins hinders the development of *Schistosoma*, and the knockdown of adenylate kinase enhances the apoptosis of schistosomula and could be a potential target for schistosomiasis prevention [55].

We then selected epitopes with higher MHC-binding affinity scores, as these are more likely to be recognized and processed by B cells. This feature is vital to the vaccination strategy, as neutralizing antibodies play a key role in targeting the pathogen, whereas helper T cells support long-term antibody production and sustain CTL activation for an effective immune response. Furthermore, the selected epitopes were screened for overlapping patterns to develop a pan vaccine against the three *Schistosoma* species. However, the emphasis on AK2, a single adenylate kinase protein, limited the number of predicted B-cell epitopes. Additionally, the exclusion of surface antigens, such as tetraspanins and glutathione-S-transferases, may have narrowed the antigenic scope. Future studies will incorporate these proteins to enhance B-cell epitope diversity and immunogenic coverage. The moderate population coverage reflects the restricted HLA distribution of the selected epitopes, which were intentionally derived from conserved AK2 protein sequences. The study provides important proof-of-concept evidence for exploiting kinases as vaccine targets in *Schistosoma*. Given their functional essentiality and conserved nature, kinases represent attractive antigens for rational vaccine design. While this strategy ensures functional relevance and cross-species conservation, it may reduce coverage in regions with diverse HLA backgrounds, such as Africa and Asia. To overcome this limitation, subsequent iterations will broaden the HLA allele selection by incorporating Class I and II epitopes common in endemic populations to improve regional and global applicability. Vaccine was constructed by using linkers. Linkers in multi-epitope vaccines prevent junctional antigen formation, ensuring proper epitope recognition and an appropriate immune response. They also enhance antigen processing and presentation, leading to efficient MHC binding and a stronger immune response. The linkers were chosen based on structural stability, rigidity, immunogenicity, and target sites for proteosomes. Studies on adjuvants used with the most promising schistosomiasis vaccine antigens have shown that they effectively enhance CD4+ T-cell responses and IFN-γ production, both of which are crucial for protective immunity against parasitic infections. Considering these observations, we chose 50S ribosomal protein L7/L12 as an adjuvant for the multi-epitope vaccine. These adjuvants have been shown to induce dendritic cell (DC) maturation, resulting in the activation of CD4+ and CD8+ IFN-γ-secreting cells. In our study, the epitopes in the structure of the multi-epitope protein were IFN-γ producing, suggesting their potential to induce immunogenic responses. However, we acknowledge that the role of L7/L12 in inducing a Th1-skewed immune response in schistosomiasis remains unvalidated. Given that effective anti-helminth immunity relies on robust Th1 polarization, further in vitro and in vivo studies are warranted to confirm whether L7/L12 is sufficient or if a combination with other TLR agonists may be necessary to achieve the desired immunological profile. Similar considerations regarding adjuvant efficacy and immune coverage have been addressed in recent SARS-CoV-2 vaccine development efforts [56]. In addition, the C-ImmSim server predicted the formation of appropriate immunoglobulins following immunization to combat *Schistosoma* infection. The results highlight the potential of our vaccine candidate to trigger an effective immune response that may offer protection against diseases. However, we recognize that these simulations are theoretical and do not fully account for the complexity of host–parasite interaction. In particular, schistosomes deploy a range of immune evasion mechanisms, such as tegument turnover, antigenic variation, and immune modulation, which are not modelled by C-ImmSim. These *in silico* results are preliminary and serve as hypothesis-generating insights. They require validation through in vivo studies using appropriate disease models.

Helminths have developed sophisticated strategies to modulate host immunity, often through interactions with pattern recognition receptors (PRRs), such as Toll-like receptor 4 (TLR4). Durães et al. demonstrated that co-culturing bone marrow-derived dendritic cells (BMDCs) with *S. mansoni* schistosomula tegument (Smteg) leads to the upregulation of co-stimulatory molecules and the production of cytokines, including IL-12p40 and TNF-α. These responses were TLR4 dependent, indicating that helminth-derived antigens can activate immune signaling cascades through direct engagement with TLR4. In our study, protein–protein docking analysis revealed strong intermolecular interactions between the multi-epitope vaccine construct and human TLR4, including a substantial number of hydrogen bonds that contributed to complex stability. Molecular dynamics simulations further confirmed the structural integrity of the vaccine–TLR4 complex over a 300-ns trajectory. The RMSD and Rg values remained stable, indicating a compact and well-folded complex structure. RMSF analysis revealed minimal atomic displacement, with binding site residues fluctuating below 0.5 Å. This indicates low structural deviation and stable interactions. Additionally, the free binding energy values were negative, confirming the high binding affinity between the vaccine and TLR4. Lower RMSD and Rg values, as supported by previous studies, are indicative of increased structural stability, which reinforces the reliability of the vaccine–TLR4 interaction model. Notably, the incorporation of the 50S ribosomal protein L7/L12, a well-characterized TLR4 agonist, at the N-terminus of the vaccine construct is expected to further potentiate TLR4-mediated immune activation. This strategic design enhances the immunostimulatory potential of the vaccine, complementing the natural ability of helminth antigens to engage with TLRs [20].

Further exploration of suitable expression vectors is essential to enable the scalable production of the vaccine construct, which is a critical step toward ensuring its practical application and large-scale availability. In this study, in silico cloning was employed to evaluate the effectiveness of the designed vaccine sequence in a prokaryotic host system, specifically *E. coli*. The widely used *pUC19* plasmid vector was selected for this purpose, and the vaccine sequence was successfully inserted into it. The simulation results indicated that the construct could be efficiently expressed within the host, suggesting its potential to activate the immune system and trigger protective immune responses. These findings support the feasibility of using this approach for preliminary expression analysis prior to laboratory-based validation [33].

Although in silico immune simulations are invaluable for directing experimental protocols, they play a key role in optimizing research efficiency and conserving resources. Computational predictions must be supported by experimental validation to confirm their biological relevance in vivo. The next essential step is to undertake preclinical studies aimed at verifying the immunogenic potential of the proposed vaccine. This includes comprehensive immunological assays to assess immune response profiles and functional activity in both in vitro and in vivo systems. In this context, we draw attention to the rEGVac vaccine study against *Echinococcus granulosus*, which successfully translated immunoinformatics into experimental animal validation, thereby demonstrating the importance of bridging computational and laboratory phases [57]. A robust multitier strategy, such as that implemented in the TgVax452 vaccine study for *Toxoplasma gondii*, offers an exemplary framework by integrating computational modelling, molecular dynamics-driven analysis of immune receptor interactions, and experimental testing to verify immunogenicity [58]. Adopting such an approach in future studies will help bridge the translational gap in our pipeline by enabling a more precise simulation of innate immune responses and facilitating the in vitro and in vivo validation of vaccine efficacy. In addition, we acknowledge that our current study did not incorporate a network-based proteome screening approach, which leverages protein–protein interaction networks and functional prioritization. Integrating such methodologies, as demonstrated in proteome-wide reverse vaccinology studies on pathogens such as *Staphylococcus aureus*, could enhance systematic antigen discovery and support the development of multi-antigen constructs with broader population coverage [59]. Future studies will incorporate both network-based antigen prioritization and experimental validation to strengthen vaccine candidate selection. Furthermore, both in vitro and in vivo experiments are necessary to evaluate the safety, efficacy, and broader immunological impact of these treatments, thereby strengthening the translational value of the findings.

## Conclusion

The *Schistosoma* AK2 protein was subjected to computational methodologies to delineate T-cell and B-cell epitopes, which were subsequently employed to engineer a multi-epitope vaccine against schistosomiasis in this study. Our comprehensive *in silico* analysis suggests that the schistosomiasis vaccine construct is highly immunogenic, safe, nontoxic, and thermodynamically stable. The designed vaccine candidate will require further refinement and experimental validation through in vitro and in vivo assays to confirm its preliminary safety, immunogenicity, and efficacy. Additionally, we acknowledge the absence of network-based proteomic screening in this study. Incorporating such analyses in future studies could strengthen antigen selection by identifying functionally relevant targets with broader population coverage.

## Supplementary Information

Supplementary Material 1: Supplementary Fig. 1. Molecular dynamics simulation study of TLR4 A) Root mean square deviation B) Root mean square fluctuations C) Radius of gyration D) Solvent-accessible surface area for a time duration of 300 ns. Supplementary Table 1: Molecular Dynamics Simulation Parameters of TLR4. Supplementary Fig. 2. Molecular dynamics simulation study of Vaccine A) Root mean square deviation B) Root mean square fluctuations C) Radius of gyration D) Solvent-accessible surface area for a time duration of 300 ns. Supplementary Table 2: Molecular Dynamics Simulation Parameters of Vaccine.

## Data Availability

Structural data that is generated within the manuscript will be available if required from the corresponding author(s).
