# Supplementary material for: In silico design of a multi-epitope pan vaccine targeting Schistosoma species
Source: Genomics Inform. 2025 Oct 27;23:21. doi: 10.1186/s44342-025-00053-4 (PMC12560484; doi:10.1186/s44342-025-00053-4)
Supplement: Supplementary file 1 — Supplementary Material 1: Supplementary Fig. 1. Molecular dynamics simulation study of TLR4 A) Root mean square deviation B) Root mean square fluctuations C) Radius of gyration D) Solvent-accessible surface area for a time duration of 300 ns. Supplementary Table 1: Molecular Dynamics Simulation Parameters of TLR4. Supplementary Fig. 2. Molecular dynamics simulation study of Vaccine A) Root mean square deviation B) Root mean square fluctuations C) Radius of gyration D) Solvent-accessible surface area for a time duration of 300 ns. Supplementary Table 2: Molecular Dynamics Simulation Parameters of Vaccine. [file 44342_2025_53_MOESM1_ESM.docx]

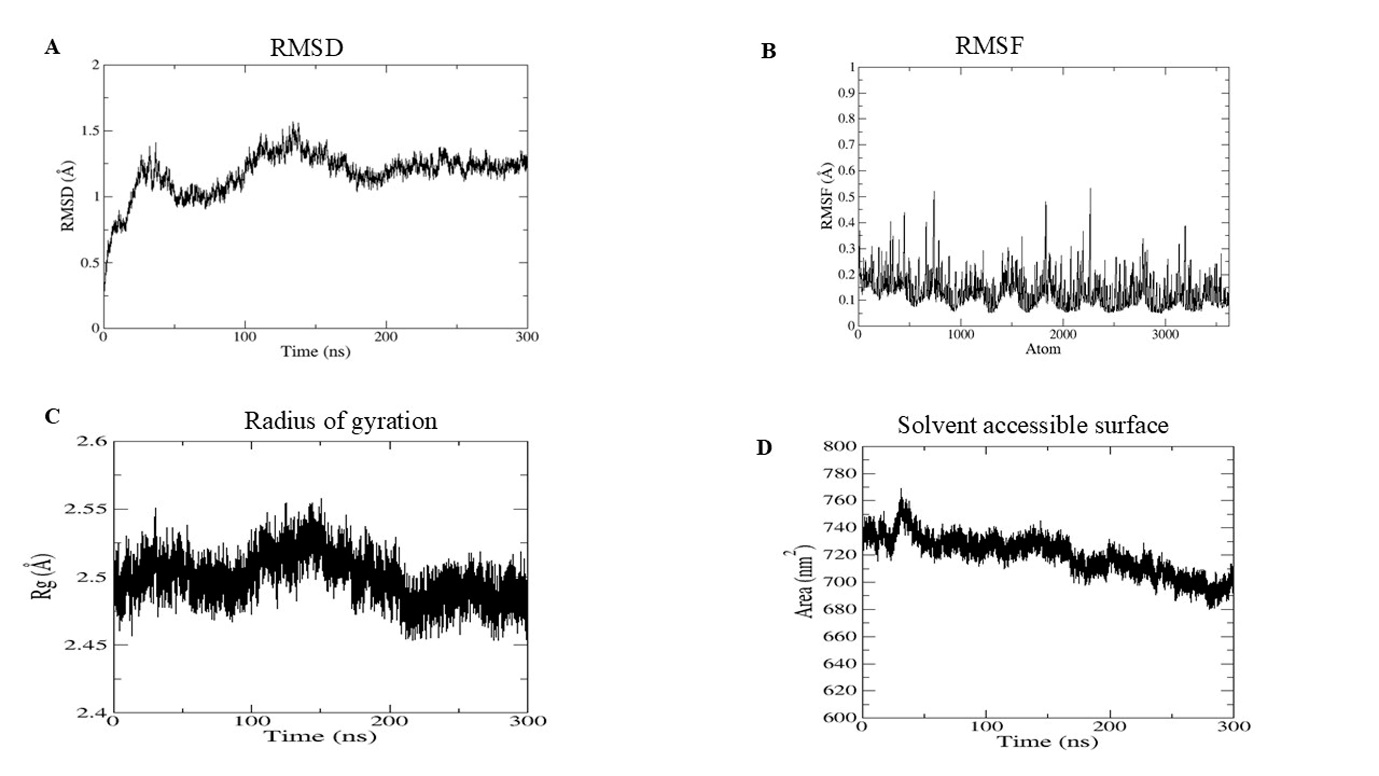


**Supplementary Figure 1.** Molecular dynamics simulation study of TLR4 A) Root mean square deviation B) Root mean square fluctuations C) Radius of gyration D) Solvent-accessible surface area for a time duration of 300 ns.

**Supplementary table 1: Molecular Dynamics Simulation Parameters of TLR4**

| **TLR4** | **Average** | **SEM** |
| --- | --- | --- |
| RMSD (Å) | 1.177 | 0.0009 |
| RMSF (Å) | 3.749 | 0.0081 |
| GYRATE (Å) | 5.651 | 0.0091 |
| SASA (nm^2^) | 718.591 | 0.0839 |


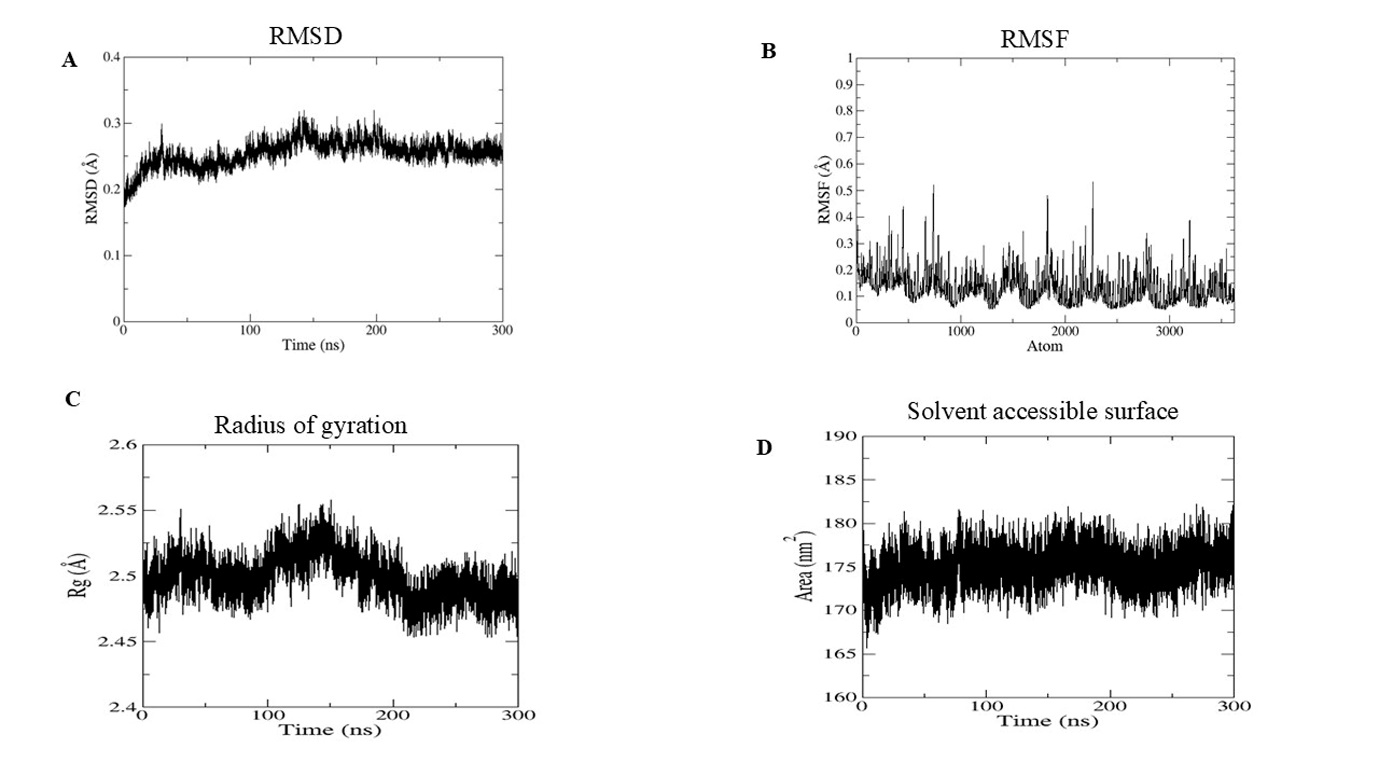


**Supplementary Figure 2.** Molecular dynamics simulation study of Vaccine A) Root mean square deviation B) Root mean square fluctuations C) Radius of gyration D) Solvent-accessible surface area for a time duration of 300 ns.

**Supplementary table 1: Molecular Dynamics Simulation Parameters of Vaccine**

| **VACCINE** | **Average** | **SEM** |
| --- | --- | --- |
| RMSD (Å) | 0.254 | 0.0001 |
| RMSF (Å) | 0.140 | 0.0008 |
| GYRATE (Å) | 2.498 | 9.1364E-05 |
| SASA (nm^2^) | 175.091 | 0.0111 |
